# Polycystic ovary syndrome among Indian adolescent girls – A systematic review and metanalysis

**DOI:** 10.3126/nje.v11i3.38460

**Published:** 2021-09-30

**Authors:** Megha Sharma, Meenakshi Khapre, Vartika Saxena, Pawna Kaushal

**Affiliations:** 1,4 MPH PG School of Public Health AIIMS Rishikesh, Uttarakhand, India; 2,3 Deptartment of Community and Family Medicine, AIIMS Rishikesh, Uttarakhand, India

**Keywords:** Adolescence, Epidemiology, Menstrural disorder, Polycystic ovaries, Prevalence

## Abstract

**Background:**

Polycystic ovary syndrome (PCOS) is a common endocrine disorder in the progenitive age group and the leading cause of infertility. The worldwide prevalence of PCOS in women varies between 2.2% to 26%. Due to limited literature on burden of PCOS among adolescent girls, its significance is still unfathomed as a research is few and far between in the present time. We conducted Systematic review and metanalysis to estimate the pooled prevalence of PCOS among Indian adolescent girls (14-19 years).

**Methods:**

With the help of a search strategy, two authors searched Scopus, Embase and Pubmed independently. We screened studies considering eligibility criteria and extracted data. Selected studies were assessed for quality and risk biases using the NIH tool. R software was used for analysis.

**Results:**

Twelve studies were included in the meta-analysis. The total number of participants in the study was 4473. All studies scored average and above as per the NIH quality assessment tool. The prevalence of PCOS among adolescents based on the Rotterdam criteria was 17.74 per 100 (CI = 11.77-23.71) with I^2^ =97 %. Hospital-based studies had a comparatively higher prevalence of PCOS as compared to community-based.

**Conclusion:**

Pooled prevalence of PCOS among Indian adolescents’ girls was high, approximately one in five.

## Introduction

Polycystic ovary syndrome (PCOS) is a common endocrinological problem in the progenitive age group and the leading cause of infertility. PCOS expose women at high risk of developing complications, such as infertility, metabolic disorders such as type 2 diabetes mellitus, hypertension, other cardiovascular and cerebrovascular disorders, if it remains untreated [1]. Such complications impair social and mental well-being, and adversely impacted the quality of life of the patients. Long-term follow-up of 786 women with PCOS observed an elevated risk of endometrial cancer [2,3]. The prevalence of PCOS ranges from 2.2% to 26% worldwide [4]. Among Asian countries, the prevalence of PCOS ranges from as low as 2.4% in China [5] to 19.5% in Iran [6]. A recent metanalysis (2019) by Naz et al. concluded that there is a regional variation in the prevalence of PCOS among adolescents. Varying prevalence is due to different diagnostic criteria, i.e. National Health Institute (NIH) 1990, Rotterdams (2003) and Androgen excess society (AES) 2006. The reported prevalence was 11.04% with Rotterdam criteria, 3.39% with NIH criteria, and 8.03% based on AES [7].

The World Health Organization (WHO) defines an adolescent as any person between 10 and 19 years. Due to the modern lifestyle, PCOS is commonly seen in the adolescent age group. The clinical manifestation of PCOS may be complete in adolescence, but the diagnosis is challenging as some features of PCOS overlap with the transitional characterstics from puberty to adulthood [8]. Hyperandrogenism and oligoanvolution criteria suggested by AES (2006) is not considered valid for early teenagers [9].

To date, available studies possessess limited sample size which failed to capture attention of policy makers. School health programme could have been the best platform to capture PCOS at an early age. Due to limited awareness of this issue among the school staff, health care workers and beneficiaries, prevailing cultural taboos, this issue remain unaddressed, neglecting its long term effect on women’s physical and mental health. This systematic review and meta-analysis aimed to estimate the pooled prevalence of PCOS among adolescent girls (14-19 years) in India. Mean age of girls in India to start menstruration is 12 years and PCOS occurs after 2 years of menarche, therefore we chosen 14-19 years age group adoloscents.

## Methodology

### Literature Searches

This review’s conducting and reporting adhered to the PRISMA (Preferred reporting items for systematic reviews and meta-analyses) guidelines [10]. We extensively searched studies from 2011 onwards, three databases (PubMed, Embase, and Scopus) with Mesh terms related to polycystic ovarian syndrome and adolescence like subheadings B.M.I, Oligomenorrhea, Hirsutism, PCOS, Waist-Hip ratio. The search strategy for each database is in Annexure 1.

### Inclusion and exclusion criteria

The observational studies with the following criteria: (1) Cross-sectional or cohort studies with participants as Indian Adolescent girls (14-19 years). (2) Outcome measured in terms of PCOS prevalence using NIH Criteria, AES criteria or Rotterdam’s criteria. We extracted the data of age group 15-19 years from the study. However while searching databases we found studies related to age group 10-19 years. Onset of menarche more than 2 years was inclusion criteria for this studies and considering the mean age of menarche as 12, we decided to include this studies. There may be very negligible proportion below 14 years. We excluded the article with non-English language and non-availability of full text with our best efforts.

### Selection of studies and Data Extraction

We exported eligible studies to Zotero version 5 and removed duplicates. Two researchers (MK and MS) trained in Systematic review independently screened each article using a three-step approach. First, we reviewed the titles and abstracts of studies and excluded studies that did not meet eligibility criteria. We further examined the retrieved full text of eligible studies and noted the reasons for exclusion. Discrepancy was resolved with discussion and a third researcher (VS). We Cross-referenced all included studies to find out missed out relevant studies, searched through Google Scholar.

MK and MS independently extracted data with data extraction form. Data extraction form consisted of study design, sample size, place of study(urban/ rural), Setting (community/school/hospital), publication year, sampling technique, diagnostic techniques, and PCOS prevalence. We resolved the discrepancy in data extraction by discussion and built consensus.

### Risk of Bias

We used the NIH quality assessment tool [11] to assess the quality and risk of bias of the included studies. The NIH tool consisted of 14 dichotomous (yes/no) questions, out of which Q.10 and Q.13 were not applicable for cross-sectional studies. We rated the quality of each study based on our best judgment. If the answer to any question was “No”, then examined the potential of bias in a study.

### Data Analysis and Synthesis

Data management was done using Microsoft Excel 2013. We performed a narrative synthesis of included studies. Metaanalysis (pooled prevalence of PCOS) and forest plots was plotted with R software version 3.6.1. Clinical, methodological and statistical heterogeneity among studies was assessed. For statistical heterogeneity, chi-square, p-value <0.05 and I2 >40% was considered. The random-effect model (Dersermonion and Laird method) with “dmetafor” function was used for analysis due to high heterogeneity. We assessed and quantified the risk of publication bias with the Funnel plot and Eggers regression test, respectively. We also found outlier using “dmetar” package in R. We performed a Sensitivity analysis to explore the influence of study setting and study design on PCOS prevalence. We have merged school/colleges as community-based studies for sensitivity analysis.

This metaanalysis was registered at Prospero (Registration# CRD42020212443). Ethical approval was obtained from Institutional Ethical Committee prior to commencing the systematic review.

## Results

**Figure 1:**
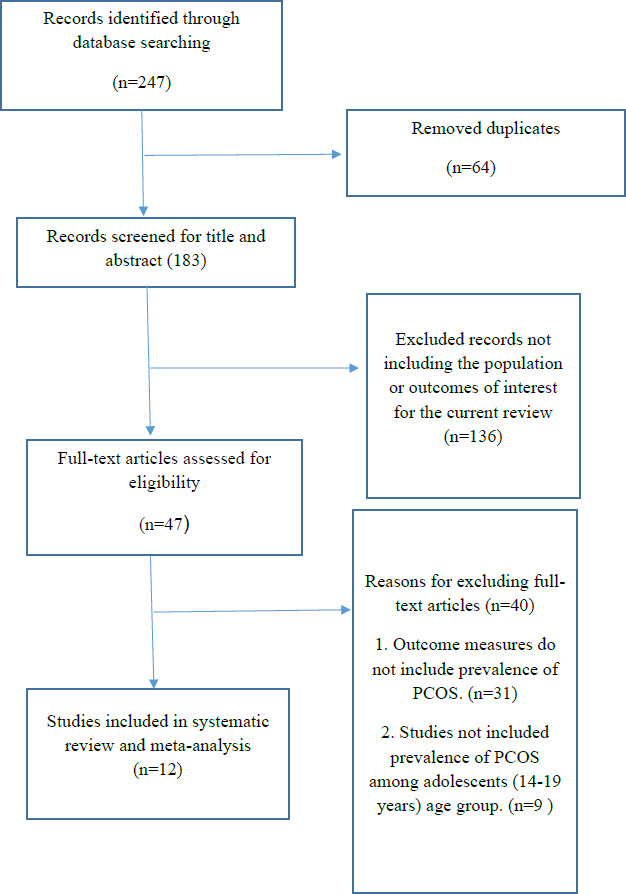
PRISMA flow diagram which included database search using keywords, title, abstract screening and full text

The extensive search of potential studies in the three databases, yielded a total of 247 studies which were imported to Zotero. After removing the duplicates (n=64) and relevant 47 studies were subjected to full-text review, and finally included 12 articles in the present systematic review and meta-analysis. (Fig 1)

### Characteristic of included studies

Characteristics of included studies is given in table 1. In 12 studies, 528 adolescents’ girls (14-19 years) were having PCOS out of 3,945 girls. The mean age of the participants was 16.8 (2.059) years. Four studies were published between 2011 and 2014, five studies between 2015 to 2018 and three studies between 2019 and 2020. Seven studies were conducted in the south zone, three in west, one each in east and central zone of India. Nine studies used only Rotterdam criteria, two studies used both NIH and Rotterdam and one study used AES and Rotterdam. Except one, all 13 studies were cross-sectional in nature. Six studies conducted in a hospital area, four in school/colleges and two in the community.

### Quality assessment

Cross-sectional studies scored between 6-10 out of 12, and cohort study scored 11 out of 14. Half of the studies (n=7) rated as fair quality and the later half as good (n=5). Except for three studies (14,15,23), all others have a low risk of bias. All studies reported a lower non-response rate. Studies done in a hospital setting had no recall bias as diagnosis mainly depends on the physician. Gupta [18] reporting of Hirsutism and androgen production was not based on the Ferryman-Gallewey model [24]. Participants selected their degree of hair growth across nine key anatomical sites based on pictographic representations, so we considered this study had a risk measurement bias. (Table 2)

**Figure 2:**
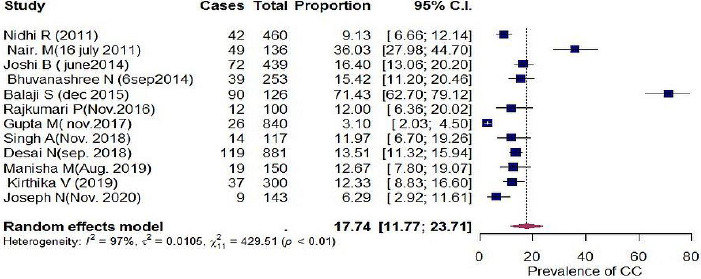
Forest plot of pooled prevalence of PCOS among adolescents (14-19 years): Random effect Model

Outcome measures and Heterogeneity among included studies We used Rotterdam crtiteria to estimate the pooled prevalence as it was used in all the included studies. Pooled proportion of PCOS among adolescents girls (14-19 years) in Indian settings with random effect model was found to be 17.74 per 100 (CI = 11.77-23.71) with I^2^ statistics of 97% (Fig 2).

### Sensitivity analysis

We conducted a sensitivity analysis to explore the influence of study setting and design on the proportion of PCOS. Figure 3 A shows pooled proportion according to different study setting. In a Community Setting with six studies, PCOS was 11% (CI=5%-17%), I2 =96%, while in a hospital setting, the pooled prevalence was 25% (CI= 12%-39%), I^2^=98%. Fig 3 B shows the pooled proportion of PCOS in cross-sectional studies was 16% (10%-22%), I^2^=97% and in one cohort study, the proportion was 36% (28%-44%).

### Publication bias

**Figure 4:**
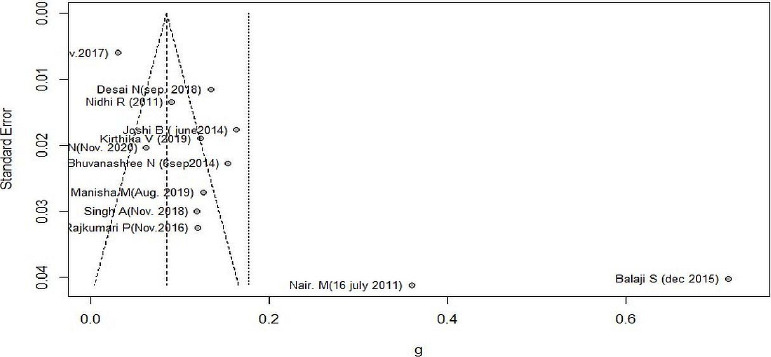
Funnel plot of included studies in meta-analysis

Fig 4 shows the funnel plot of 12 studies. Two studies Nair. M [13] and Balaji [16] are far from a funnel with large effects and small sample sizes. Gupta M. [18] had a low prevalence of 0.3 with small SE (large sample). Egger test intercept was 8.089 (3.71-12.47) and p-value < 0.05 depicting the presence of asymmetry in the funnel plot.

### Outlier analysis

**Figure 5:**
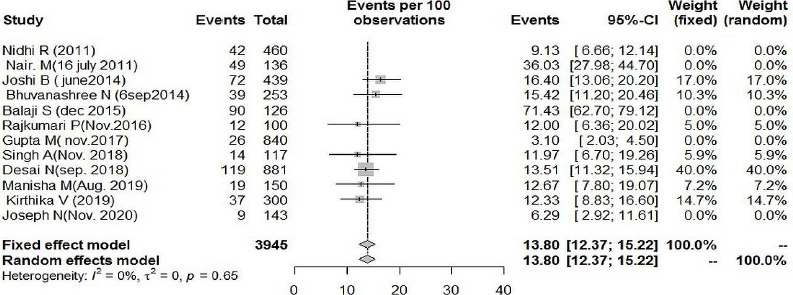
Outlier analysis of included studies

Fig 5 shows five studies as outliers, and when removed, the heterogeneity was 0 % and pooled prevalence of 13.8% CI(12.37% to 15.22%).

## Discussion

The pooled proportion of PCOS among adolescents girls age group (14-19 years) according to Rotterdam criteria in Indian settings was found to be 17.74 per 100 (CI 11.77-23.71). Balaji S et al. shows a very high proportion of PCOS, i.e. 71.43 per 100 (CI=62.70-79.12) adolescent girls [16]. Gupta M et al shows the lowest proportion of PCOS, i.e. 3.10 per 100(CI=2.03-4.50) [18]. A meta-analysis done by Naz MS 2019 [7] included studies worldwide, including four from India, five from Iran and one each from Thailand and Southern California respectively. All these studies were community-based, and the finding was comparable to our pooled community-based studies i.e 11 per 100 (0.05-0.17). The participants’ mean age in the current review was 16.8 years (SD=2.059), and in Naz was 16.99 years (16.46-17.52). The pooled prevalence as per NIH criteria was 3.39% (0.28-9.45%), and AES was 8.03% (6.24-10.01%). Publication bias was not seen in Naz study. We have not conducted a pooled analysis of prevalence as per NIH and AES criteria as it was estimated respectively in two studies and one study only [12,14,16]. Further in our meta-analysis, publication bias was seen. Two hospital-based studies [13,16] with smaller sample size had reported higher proportion of PCOS.

A meta-analysis by Ghiasi A (2019) found a lower prevalence of PCOS (7 %, 95% CI: 6-8%) among Iranian adolescents using Rotterdam criteria compared to the current metanalysis [25] Skiba AM reported prevalence of PCOS as 7 % (6-7 %) based on NIH criteria and 12% [95% CI: 10–15%] based on Rotterdam criteria [26]. Rotterdam criteria were formulated to expand NIH criteria and are a broader concept used widely in community studies. In present metanalysis after removing the outliers, the prevalence of PCOS among adolescent was 13 % per 100, that may still be under-reported. Another metanalysis by Jalilian A. et al (2015) among Iranian women (10-45 years) found a comparatively higher prevalence of PCOS (19.5 %). (6) The prevalence among women is higher than the adolescent age group because, first, PCOS is often overlooked in adolescent age. Secondly, it is challenging to diagnose as a menstrual pattern is difficult to distinguish from anovulation associated with puberty due to maturating hypothalamic pituitary ovarian axis [27].

We found a wide prevalence of PCOS within India. Meta-analysis by Ding and colleagues, reported prevalence rate in Chinese women was 5.6% (based on the Rotterdam criteria) and in Middle Eastern countries was 16% [28]. These differences in the prevalence of PCOS across geographical location, racial or ethnic groups may be attributed to environmental or genetic influence, study design, study setting, different diagnostic criteria and pattern of utilization of health care services. However, we do not attribute variation in our study to geographical location, as confirmed by Wolf, et al. [29]. Hospital based studies [12,13,16,19,21,22] may not represent the general population. Nair et al had prospectively followed a hospital-based cohort that resulted in a higher incidence of PCOS. Nidhi et al., Balaji et al., and Nair et al. were hospital-based reporting higher prevalence among five outlier studies. Other two outlier studies, Gupta et al. [18] and Joseph et al. [23], questionnaire was self administer resulting in low prevalence.

### Expert opinion

Diagnosis of PCOS in adolescence is a challenge because of overlapping symptoms of PCOS with normal pubertal changes in adolescents. Incidence of PCOS among adolescence has been increasing due to modern lifestyle. Early diagnosis is important to instill early lifestyle modifications which will prevent metabolic and reproductive complications. Health education and screening for PCOS need to be incorporated in School or community based adolescent health programs. An assessment of target groups by simple menstrual history could detect possible PCOS during early adolescence to facilitate appropriate early intervention. We also recommend open discussion on menstruation problems in adolescent groups should be encouraged in schools and aaganwadi centres. Health talk should be established between mother and daughter and break the hesitance to seek timely medical advice.

## Limitation of the study

The main limitation of our review is searching only three databases for screening and identifying studies. However, we conducted, a thorough search of the cross-references to include any eligible studies via google scholar. Epidemiology study design, varied study population and methodology added to heterogeneity.

We could find only six community-based studies with a limited sample size may not be adequate to comment on PCOS prevalence. Publication bias was noted with five outliers. Hospital-based study may have distorted the prevalence to the higher side, which was later rectified by sensitivity analysis showing comparatively lower prevalence.

## Conclusion

It can be inferred that cooking food in iron pot escalates the levels of blood hemoglobin and iron content of the food, and thus reduces the incidences of iron deficiency anemia. The bioavailability of food containing heme iron increases more when cooked in iron pot than food having non-heme iron form. Also, the content of iron in the food was found to be increased by cooking acidic food with iron ingot. Very limited research trials are available on this topic that warrants a careful interpretation of results inferred and a considerable need of larger population-based studies and randomized controlled trials for better outcomes.

## Figures and Tables

**Figure 3A:**
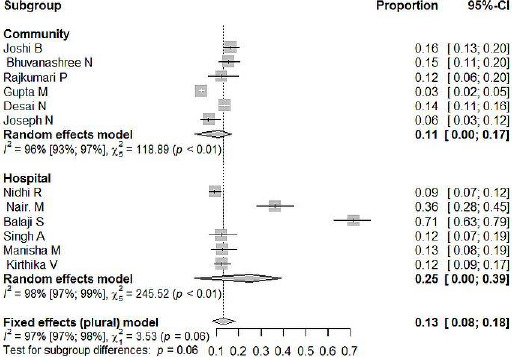
Forest plot of pooled proportion of PCOS among adolescent’s (14-19 years) according to different study setting

**Figure 3B:**
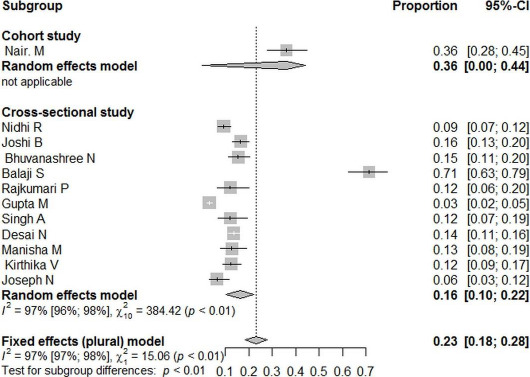
Forest plot of pooled proportion of PCOS among adolescent’s girls (14-19 years) in different study design

**Table 1:** Characteristics of the included studies

S.No.	Study	Year	Area of study	Type of study	Location	No. of participants	Age group of participant	Prevalence of PCOS		
								NIH criteria	AES criteria	Rotterdam’s criteria
**1**	**Nidhi R, et al . [12]**	2012	Semi-urban and rural area	Cross sectional	Bangalore	460	15-18 years	12	NE	42
**2**	**Nair.M, et al. [13]**	2012	Urban	Cohort study	Thiruvananthapuram	136	15-17 years	NE	NE	49
**3**	**Joshi B, et al. [14]**	2014	Urban	Cross sectional	Mumbai	439	15-19 years	NE	34	72
**4**	**Bhuvanashree,N et al. [15]**	2014	Rural	Cross sectional	Andhra Pradesh	253	10-19 years	NE	NE	39
**5**	**Balaji S, et al. [16]**	2015	Urban and Rural	Cross sectional	Tamil Nadu	126	14-19 years	31	NE	90
**6**	**Rajkumari P, et al. [17]**	2016	Urban	Cross sectional	Bhubaneswar	100	14-17 years	NE	NE	12
**7**	**Gupta M, et al. [18]**	2017	Urban	Cross sectional	Bhopal	840	15-18 years	NE	NE	26
**8**	**Singh A, et al. [19]**	2018	Urban	Cross sectional	Hyderabad	117	15-19 years	NE	NE	14
**9**	**Desai NA, et al. [20]**	2018	Urban	Cross sectional	Ahmedabad	881	13-18 years	NE	NE	119
**10**	**Manish MM, et al. [21]**	2019	Urban	Cross sectional	Maharashtra	150	10-19 years	NE	NE	19
**11**	**Kirthika SV, et al. [22]**	2019	Urban	Cross sectional	Chennai	300	14-18 years	NE	NE	37
**12**	**Joseph N, et al. [23]**	2020	Urban	Cross sectional	Mangalore	143	18-19 years	NE	NE	9
					Total	4473				

*NE=Not estimated

**Table 2:** Score based on NIH quality assessment tool, quality and biases in included studies.

S.No.	Study	Year	Score	Quality	Selection bias	Information bias	Confounding bias	Measurement bias	Non response bias	Overall risk of bias
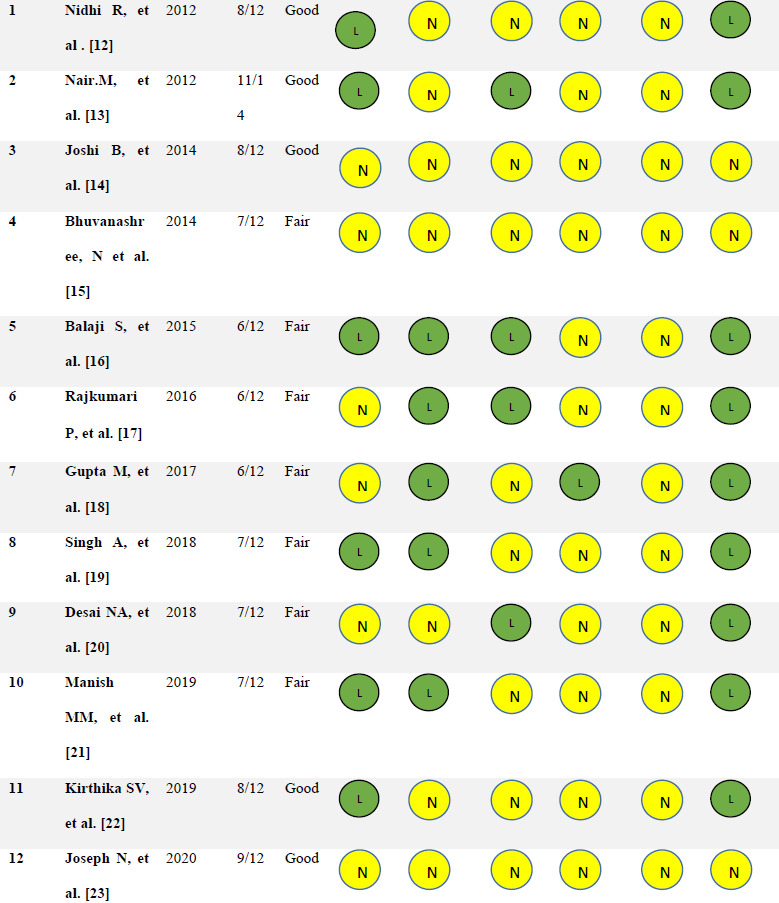										

## Appendix

**Annexure1:** Detailed search strategy used for each database

S.No.	Database	Search terms	Number of studies identified
**1**	PubMed	(((India*)) AND ((“polycystic ovarian syndrome” (MeSH) OR PCOD OR PCOS OR “Stein Leventhal Syndrome” OR “polycystic ovarian disease”))) AND (prevalence OR proportion) Filters: Abstract, Free full text, Female	16
**2**	Embase	‘ovary polycystic disease’/exp OR ‘ovary polycystic disease’ OR PCOS OR pcod AND ‘India’/exp OR India AND ‘Prevalence’/exp OR Prevalence OR proportion AND (‘clinical study’/de OR ‘cohort analysis’/de OR ‘comparative study’/de OR ‘correlational study’/de OR ‘cross sectional study’/de OR ‘human’/de OR ‘multicenter study’/de OR ‘observational study’/de OR ‘prospective study’/de OR ‘questionnaire’/de OR ‘retrospective study’/de OR ‘structured questionnaire’/de) AND [female]/lim AND (‘Article’/it OR ‘Article in Press’/it OR ‘Conference Abstract’/it OR ‘Conference Paper’/it) AND ([adolescent]/lim OR [adult]/lim OR [middle aged]/lim OR [young adult]/lim)	190
**3**	Scopus	(TITLE-ABS-KEY (india* ) AND TITLE-ABS-KEY (polycystic AND ovarian AND disease OR pcos OR pcod ) AND TITLE-ABS-KEY (prevalence OR proportion ) ) AND (LIMIT-TO (PUBSTAGE, “final” ) OR LIMIT-TO (PUBSTAGE, “aip” ) ) AND (LIMIT-TO (ACCESSTYPE(OA) ) OR LIMIT-TO (ACCESSTYPE(OTHER) ) ) AND (LIMIT-TO (AFFILCOUNTRY, “India” ) ) AND (LIMIT-TO (DOCTYPE, “ar” ) OR LIMIT-TO (DOCTYPE, “cp” ) ) AND (LIMIT-TO (LANGUAGE, “English” ) )	41
